# Synthesis, Characterization, Tautomeric Structure and Solvatochromic Behavior of Novel 4-(5-Arylazo-2-Hydroxystyryl)-1-Methylpyridinium Iodide as Potential Molecular Photoprobe

**DOI:** 10.3390/ma9121022

**Published:** 2016-12-19

**Authors:** Farag Altalbawy, Elham Darwish, Mohamed Medhat, Sayed El-Zaiat, Hagar Saleh

**Affiliations:** 1 Department of Measurements and Environmental Applications, National Institute of Laser Enhanced Sciences (NILES), Cairo University, Giza 12613, Egypt; f_altalbawy@yahoo.com; 2 Department of Chemistry, University College of Duba, Tabuk University, Duba 71911, Saudi Arabia; 3 Department of Chemistry, Faculty of Science, Cairo University, Giza 12613, Egypt; 4 Department of Physics, Faculty of Science, Ain Shams University, Cairo 11566, Egypt; mmedhat61@hotmail.com (M.M.); eelzaiat@hotmail.com (S.E.-Z.); hagarmustafa1991@yahoo.com (H.S.)

**Keywords:** arylazomerocyanine, acidity constants, azo-hydrazonetautomerism, solvatochromism

## Abstract

A novel series of the title compound 4-(5-arylazo-2-hydroxystyryl)-1-methylpyridinium iodide **6** has been synthesized via condensation reactions of the arylazosalicylaldehyde derivatives **4a**–**i** with 1-methyl-picolinium iodide **5**. The structures of the new arylazo compounds were characterized by ^1^H NMR, IR, mass spectroscopy, as well as spectral and elemental analyses. The electronic absorption spectra of arylazomerocyanine compounds **6** were measured in different buffer solutions and solvents. The pK′s and pK*′s in both the ground and excited states, respectively, were determined for the series and their correlations with the Hammett equation were examined. The results indicated that the title arylazomerocyanine dyes **6** exist in the azo form **6A** in both ground and excited states. The substituent and solvent effects (solvatochromism) of the title compound arylazomerocyanine dyes were determined using the Kamlet-Taft equation and subsequently discussed.

## 1. Introduction

Many arylazo compounds are represented in important classes of widely studied colored organic compounds. They are used in a variety of industrial applications including laser materials, ink-jet inks, disperse dyes, and in reprographic technology, laser printing and hair dyeing [1,2,3,4,5]. Also, arylazo function groups are important in progressing antineoplastics activity [6,7]. In addition other arylazo dyes have also attracted much attention due to their diverse photodynamic therapy, as lasers and second order non-linear optical materials (NLO) suitable for applications such as optical switching and harmonic generation [8,9,10].

Merocyanine or styryl dyes have been utilized as sensitizing additives in color photography [11], as photoelectrodes in dye-sensitized light-weight plastic solar cells [12], and as molecular probes to measure the potential of the membrane in biological systems [13]. Also merocyanines are well known to be sensitive to the polarity of solvents so these dyes show very interesting spectral, photochemical behavior and solvatochromic properties [14], as well as being photoreactive compounds sensitive to the acidity of the medium [15], so that in acidic or basic medium these dyes can undergo protonation or deprotonation reactions [16]. Due to these properties, these dyes are very attractive for numerous photonic applications [17] such as laser phototherapy [18], molecular electronics [19], nonlinear optics [20], solar photovoltaic′s [21] and laser materials [22].

**Scheme 1:**
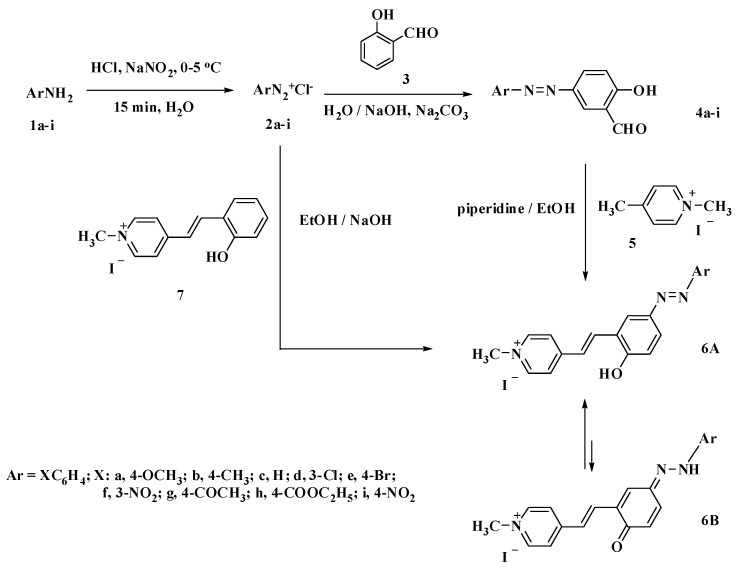
Synthesis of arylazosalicylaldehyde **4a**–**i** and 4-(5-arylazo-2-hydroxystyryl)-1-methylpyridinium iodide **6a**–**i**.

In the light of the above findings we have continued our efforts to synthesize and study spectrophotometrically arylazo derivatives of heterocyclic compounds and merocyanine dyes to explore their applications as photoprobes, and for pH sensing and water sensing in organic solvents [23,24,25,26,27,28]. We display here our interest in the synthesis of 4-(5-arylazo-2-hydroxystyryl)-1-methylpyridinium iodide using and applying the physical constraints approach to evaluate its acid dissociation constant to elucidate the actual tautomeric structure in both its ground and excited states via correlated pK′s and pK*′s by the Hammett equation. To the best of our knowledge there have been no reports hitherto on the synthesis of the latter derivatives. Furthermore, it was thought interesting to elucidate the actual tautomeric structure of the compounds in question (Scheme 1) and determine their solvatochromic properties via application of the Kamlet-Taft equations with discussion prior to investigating their biological activities and applications as potential photoprobes.

## 2. Results and Discussion

### 2.1. Synthesis and Characterizations

The required series of arylazosalicylaldehyde **4d**–**f**,**h** were prepared by selective coupling of the salicyladehyde with the appropriate diazotized aniline in 80% ethanol-water mixture in the presence of a NaOH and Na_2_CO_3_. This coupling reaction was accomplished selectivity at the position-5 of the salicyladehyde ring as outlined in (Scheme 1). The structures were confirmed by elemental analyses and spectral (Infrared spectra (IR), ^1^H NMR and mass spectrum (MS)) data. For example, the IR spectrum of arylazo compound **4h** (see Experimental Section), taken as a typical example of the series arylazosalicylaldehyde, revealed three stretching bands at ν_max_ = 3435, 1706, and 1657 cm^−1^ for the OH, ester C=O and aldehyde C=O groups, respectively. The ^1^H NMR spectrum revealed two triplet signals at δ 1.35 and a quartet at 4.31 ppm attributable to the CH_3_ and CH_2_ of the ester group, respectively, and another two singlet signals one of them at δ 10.37 ppm assignable to CHO group and the other a broad singlet at δ 11.61 ppm assignable to the OH group, in addition to the aromatic protons. A molecular ion peak in the mass spectrum at *m*/*z* 298 was apparent which is in agreement with the molecular formula C_16_H_14_N_2_O_4_. The other arylazosalicylaldehydes **4a** [29] and **4b**,**c**,**g**,**i** [30] were prepared as previously reported.

Next, refluxing equimolar amounts of the appropriate arylazosalicylaldehyde **4a**–**i** and 1-methyl-4-methylpyridinium iodide **5** in EtOH containing catalytic amounts (few drops) of piperidine as basic catalyst, afforded in each case one isolable product (as evidenced by TLC analysis of the crude product) which was identified as the 4-(5-arylazo-2-hydroxystyryl)-1-methylpyridinium iodide derivatives **6a**–**i** formed in good yields 76%–95% yield as outlined in (Scheme 1). The structure of compounds **6a**–**i** were elucidated by elemental and spectral (IR, ^1^H NMR and mass) data. For example, the IR spectra revealed an absorption band in each case near ν_max_ = 3430 cm^−1^ corresponding to the stretching OH group in addition to the expected bands of aliphatic CH and aromatic C=C stretching. ^1^H NMR spectra of compounds **6** revealed in addition to the expected signals of the aromatic protons, and singlet at δ = 4.09–4.26 ppm a signal of the protons of one methyl group (NCH_3_), a singlet at δ = 6.25–6.77 ppm assigned to the OH proton. The mass spectra of all products **6** exhibited in each case, a molecular ion peak at the correct molecular weight for the respective compound (see Experimental Section).

Finally coupling equimolar amounts of the appropriate diazotized anilines and 1-methyl-4-(2-hydroxystryl) pyridinium iodide **7** [31] in ethanol in the presence of a catalytic amount of NaOH gave the corresponding products, **6g**–**i** which were identical in all respects (melting point (mp) and IR spectra) with those obtained from condensation reaction of compounds **4** with **5**.

Their mass spectral data of azo disperse dyes **6a**–**i** showed in each case the respective molecular ion peak (M^+^). Although the foregoing spectroscopic data are consistent with the assigned structures **6a**–**i**, they cannot distinguish between the two possible tautomeric structures namely, the hydroxy-azo and keto-hydrazone forms **6A** and **6B**, respectively (Scheme 1). To elucidate the actual tautomeric form to compound **6**, we studied their electronic absorption spectra in different buffer solutions.

### 2.2. Determination of Acid Dissociation Constants and Tautomeric Structure for the Prepared Azo Disperse Dyes **6a**–**i**

The UV-Vis spectra of 4-(5-arylazo-2-hydroxystyryl)-1-methylpyridinium iodide **6a**–**i** in ethanol revealed, in each case, three characteristic absorption maxima, one above 500 nm, the second in the region 484–411 nm and the third in the range of 361–345 nm (Table 1). These electronic absorption patterns are similar to those of hydroxyazo compounds [32]. On the basis of such an absorption pattern, it can be concluded that the studied compounds **6** have in solution one tautomeric form, namely the hydroxyazo tautomeric form **6A**. This conclusion was confirmed by the ^1^H NMR spectra of the studied compounds **6**. Such spectra showed the OH proton signals in the region of δ = 6.25–6.77 ppm (see Experimental Section).

**Figure 1:**
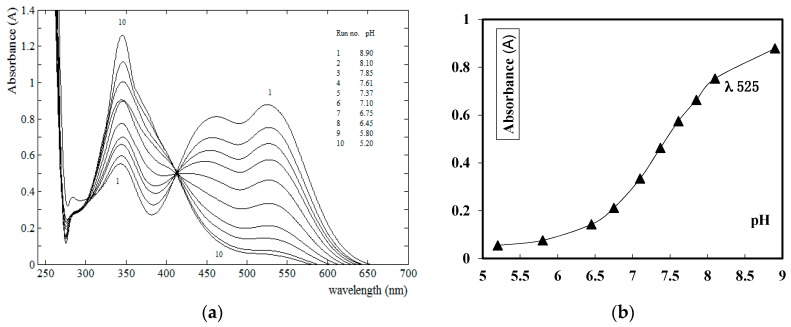
(**a**) UV absorption spectra of (**6e**), in solution of different pH values (80% dioxane-water) at 27 °C and μ = 0.10; (**b**) Spectrophotometric titration curve of (**6e**), at λ_max_ = 525 nm in 80% dioxane-water mixture at 27 °C and μ = 0.10.

To provide further evidence for the hydroxy-azo form **6A** assigned to the studied condensation and coupling products, the acid dissociation constants pKa of the synthesized series were determined and their correlation by the Hammett equation was examined [33]. The pKa values for the series **6a**–**i** were determined spectrophotometrically in 80% dioxane-water mixture (*v*/*v*) at 27 °C and μ = 0.10. In all runs the ionic strength was kept constant at 0.10. From the pH-absorbance spectra, typical absorption spectra of compound **6e** in such buffer solutions are shown in (Figure 1). The value of the pKa was calculated (See Experimental Section).

**Figure 2:**
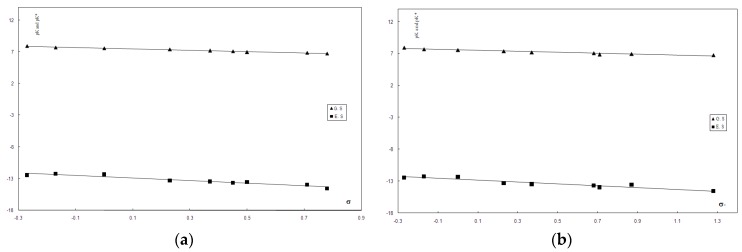
(**a**) Correlation of pK and pK* of 4-(5-arylazo-2-hydroxystyryl)-1-methylpyridinium iodide **6a**–**i** with Hammett substituent constants σ; (**b**) Correlation of pK and pK* of 4-(5-arylazo-2-hydroxystyryl)-1-methylpyridinium iodide **6a**–**i** with enhanced Hammett substituent constants σ^−^.

The acid dissociation constants pKa values determined for the compounds **6a**–**i** are listed in (Table 2). The pKa values were plotted versus the Hammett substituent constant σ_X_ and exalted Hammett substituent constant $\sigma_{x}^{-}$ using the least-squares method as shown in (Figure 2) [33] and the equations corresponding to the straight lines obtained are:

pKa = 7.57 − 1.07σ_X_, *r*^2^ = 0.983, *s* = ±0.05

$$\mathrm{pKa}=7.55-0.75\sigma_{x}^{-},r^{2}=0.932,s=\pm 0.11$$

where *r* is the correlation coefficient and *s* the standard deviation. From these values of *r* and *s*, the pKa data from **6a**–**i** seem to be better correlated with the Hammett substituent constant σ_X_ rather than the enhanced Hammett substituent constant $\sigma_{x}^{-}$. Such excellent linear correlations with σ indicate that the studied compounds exist in the 1H-structure, namely the hydroxyazo form **6A** (Scheme 1). This is because if they existed in one of the mixed hydroxyazo-ketohydrazone tautomeric forms **6A** or **6B**, their acid dissociation constants pK values would be better correlated with the exalted Hammett substituent constant σ^−^ rather than σ as the substituent would be in a direct interaction with the negative charge on the nitrogen atom in the conjugate base. This finding indicates that compounds **6a**–**i** exist in the hydroxyazo form **6A** in solution. Furthermore, the assignment of form **6A** is substantiated by the similarity of the reaction constant ρ value 1.07 for the series studied **6a**–**I** with that reported for 2-arylazophenols, ρ = 1.22 [34]. However, for a typical hydrazone, the ρ value was reported to be 2.57 [35].

Next, the acid dissociation constants pK*′s of the studied compounds in their corresponding singlet excited states were calculated by utilizing the so-called Forester energy cycle [36]. According to this energy cycle:

pK* = pK + (Δν) (0.625/*T*)

where pK and pK* are the acid dissociation constants in the ground and excited states, respectively and Δν represents the frequency difference in cm^−1^ between the values of the absorption maximum λ_max_ of the compound in acid and alkaline media [37,38,39].

The results of such calculations are summarized in Table 2. We examined the correlation of these acidity constants pK* of **6** with the Hammett substituent constant as shown in Figure 2. Plots of pK* for the series **6** vs. σ_X_ and $\sigma_{x}^{-}$ were linear. The equations of the regression lines are:

pK* = −12.70 − 2.05σ_X_, *r*^2^ = 0.92, *s* = ±0.10

$$\mathrm{pK}*=-12.71-1.45\sigma_{x}^{-},r^{2}=0.89,s=\pm 0.17$$

Such linear equations indicate that the studied series **6a**–**i** have also predominantly the hydroxyazo tautomeric form in their excited states. As shown, the reaction constants in the excited state (ρ*) obtained are larger than those of the ground state. Assuming that the atom connectivity is the same in both ground and excited states, such results emphasize the importance of the electronic interaction in the excited state [37,38,39]. Furthermore, such linear correlations together with the similarity of the ρ* values collectively indicate that the studied compounds **6** exist predominantly in the 1H-structure, namely form **6A** (Scheme 1) in their excited states too. This is only to be expected if the lifetime of the excited state before electronic relaxation is too short to allow equilibration with the other tautomeric structure.

### 2.3. Solvatochromic Behavior

**Figure 3:**
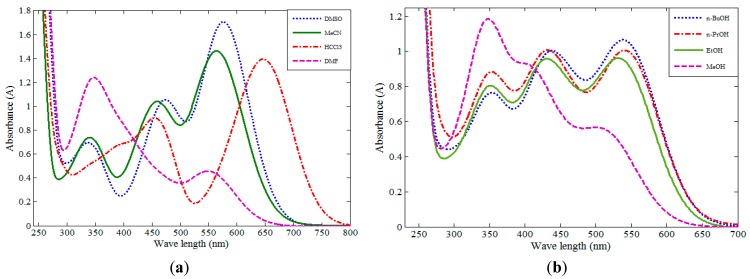
Electronic absorption spectra of 2.0 × 10^−5^ M compound **4e**: (**a**) In dimethylsulfoxide (DMSO), MeCN, HCCl_3_ and dimethylformamide (DMF); (**b**) In *n*-butanol, *n*-propanol, ethanol and methanol.

**Figure 4:**
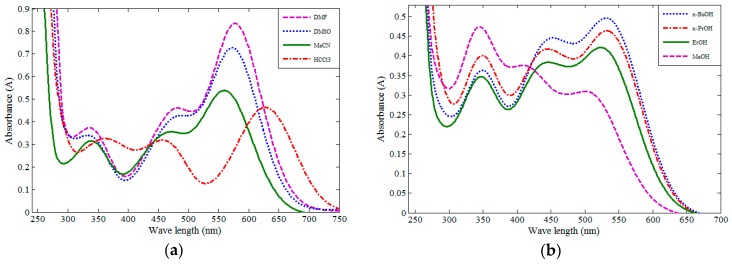
Electronic spectra of 2.0 × 10^−5^ M compound **4f**: (**a**) In DMSO, MeCN, HCCl_3_ and DMF; (**b**) In *n*-butanol, *n*-propanol, ethanol and methanol.

The electronic absorption spectra of the title compounds **6a**–**i** were recorded at concentration of 10^−5^ M over the range 300–800 nm in eight various organic solvents of different physical properties, namely acetonitrile, *n*-propanol, *n*-butanol, methanol, ethanol, chloroform, dimethylformamide (DMF) and dimethylsulfoxide (DMSO). The results of the λ_max_ bands are listed in Table 1 and Table 3. As shown in these tables, the spectra of each compound of the studied series showed that they are comprised of mainly two or three bands, depending upon the molecular structure of the substituent and the nature of the solvent. Each of the studied compounds exhibits absorption bands in the ranges 340–360 nm, 400–510 nm and 630–760 nm in all solvents used. The latter UV λ_max_ peaks have a charge transfer character, due to π-π* electron transfer during the S^0^–S^1^ transition. With increasing solvent polarity a bathochromic shift (positive solvatochromism) and a splitting of the UV–Vis absorption band, caused by hydrogen bond formation and deprotonation, is observed [40]. The main Vis band displayed by all substituted compounds in the region 630–760 nm is a highly intense one and is relatively affected by the changing of the solvent and the substituent present; such as, the absorption spectra of the two compounds **6e** in Figure 3 and **6f** in Figure 4. Table 1 and Table 3 in different solvents show a band appearing at longer λ_max_ indicating that such dyes may be liable to form a solvated complex [41,42].

Next, the effects of solvents on the absorption spectra of the synthesizedcompounds **6a**–**i** were evaluated and interpreted by use of multiliner regression analysis. The spectral shifts in different solvents, solute solvent interactions were measured by polarity/polarizability, hydrogen bonding and basicity of solvents which are considered as the main contributions to compare acidity by means of the linear solvation energy relationship (LSER), namely the Kamlet-Taft equation (Equation (1)) [43,44]:

υ = *υ*_o_ + sπ* + *b*β + *a*α (1)

where υ is the solvatochromic property of interest (wave number in the maximum of absorption band in cm^−1^), π* is the measure of solvent dipolarity/polarizability, β is the scale of the solvent hydrogen bond acceptor (HBA) basicities, α is the scale of the solvent hydrogen-bond donor (HBD) acidities, and *υ*_o_ is the solute property of a reference system and represents the regression intercept which corresponds to the gaseous state of the spectrally active substance. The solvent parameters are given in Table 4 and the values of such solvent regression factors are depicted in Table 5.

The regression coefficients s, *a* and *b* are fitted at the 95% confidence level, whose values depend on the degree of contribution of each solvent parameter (π*, β and α) to the expected values. The AS multiple correlation coefficient value reflects the goodness of the fit, the values (0.993–0.810) of the correlation coefficient indicated that the correlation between spectroscopic data and the certain solvent parameter is acceptable [45].

The negative sign of (*b*) regression coefficient indicated a bathochromic shift with regard to the increasing solvent hydrogen bond acceptor basicities and solvent dipolarity/polarizability and vice versa. This increases the stability of the resonance structure in the absorption of merocyanine dyes and decreases the reorientation of the solvents molecules around the dyes. The positive sign of the (*a*) and (*s*) coefficients for all the dyes indicated a hypsochromic shift relative to the increasing solvent hydrogen bond donor acidities. This refers to stabilization of the ground state with respect to the electronic excited state.

**Figure 5:**
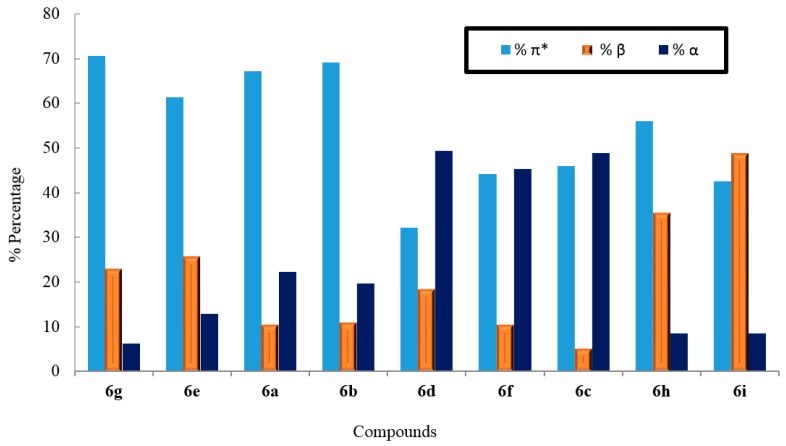
Percentage contribution of the solvatochromic parameters: π*, β and α.

From the values of regression coefficients, Table 3, the percentage contributions of all parameters to solvatochromism π*, β and α, were calculated for the studied compounds and are listed in Table 6 and showed in Figure 5.

The percentage contributions of the solvatochromic interactions, Table 6 shows that most of the solvatochromism is due to solvent dipolarity/polarizability (non-specific solvent interactions) and HBD ability (α term) which increases their electron density and thus decreases the push-pull character of the chromophore [46]. However, in cases of compounds **6g** and **6h** where β values (HBA ability) of the solvent are greater than α values, this could be explained by the interaction of HBA solvents with the carbonyl groups of the acetyl and ester groups of the **6g** and **6h** molecules, respectively. In case of compound **6i** the value of α is greater than the values of both β and dipolarity/polarizability as shown in Figure 5.

## 3. Materials and Methods

### 3.1. General Experimental Procedures

Melting points of crystalline azo compounds were measured on an electrothermal Gallenkamp apparatus and are not corrected. Solvents were generally distilled and dried by standard literature procedure prior to use. The UV-Vis spectra were recorded on a Perkin-Elmer Lambada 40 spectrometer (Perkin-Elmer, Überlingen, Germany). The IR spectra were taken with a Pye-Unicam SP300 spectrometer using KBr discs (Shimadzu, Tokyo, Japan) The ^1^H NMR (300 MHz) spectra were obtained with a Varian Mercury VXR-300 spectrometer (Varian, Inc., Karlsruhe, Germany). Mass spectra were recorded on a GC-MS Q1000-EX (Shimadzu, Tokyo, Japan) and GC-MS 5988-A HP spectrometers (Shimadzu, Tokyo, Japan), the ionizing voltage was 70 eV. Elemental analyses were carried out by the Micro analytical Center of Cairo University, Giza, Egypt. 5-arylazosalicylaldehyde **4a** [29] and **4b**,**c**,**g**,**i** [30] were prepared by literature methods and 1-methyl-4-(2-hydroxystryl)pyridinium iodide **7** [31]. Typical reaction procedures and spectroscopic data for the all new products are listed below.

### 3.2. Synthetic Procedures

#### 3.2.1. General Procedure for the Synthesis of Arylazosalicylaldehyde **4a**–**j**

A mixture of 20 mmol of an aniline derivative in concentrated hydrochloric acid (30 mL) and water (5 mL) was heated until complete dissolution and diazotized with sodium nitrite (1.4 g, 20 mmol) dissolved in little amount of water, over a period of 20 min at 0 °C. The diazonium solution was added dropwise to a solution of salicylaldehyde (2.13 mL, 20 mmol) in water (40 mL) containing sodium carbonate (7.4 g) and sodium hydroxide (0.8 g) over the course of 30 min at 0 °C. During the addition process, the solution was vigorously stirred; the reaction mixture was stirred for 30 min in an ice bath and subsequently stirred for 1 h at room temperature. The solid product was collected by filtration and washed with sodium chloride solution (10%), dried and finally crystallized from the appropriate solvent to give the respective arylazosalicylaldehyde **4a**–**i**. The physical constants of the new compounds prepared **4d**–**h** are given below.

#### 3.2.2. 5-(3-Chlorophenylazo)salicylaldehyde (**4d**)

Yellow solid; Yield: 4.43 g (85%); mp 167–168 °C (from EtOH); IR (KBr) ν_max_: 3434 (OH), 1664 (C=O) cm^−1^; ^1^H NMR (DMSO-*d*_6_): δ = 7.19 (d, *J* = 9.0 Hz, 1H, ArH), 7.42–7.46 (m, 3H, ArH), 7.62 (d, *J* = 9.0 Hz, 1H, ArH), 8.06 (d, *J* = 9.0 Hz, 1H, ArH), 8.17 (d, *J* = 6.6 Hz, 1H, ArH), 10.37 (s, 1H, CHO), 11.59 (s, 1H, OH); MS (EI, 70 eV) *m*/*z* (rel. int.): 263 (M^+^ + 3, 1), 262 (M^+^ + 2, 7), 261 (M^+^ + 1, 3), 260 (M^+^, 20), 149 (34), 139 (12), 121 (76), 113 (26), 111 (111), 93 (50), 75 (77), 65 (100), 64 (19), 63 (38), 53 (32), 50 (31); Anal. Calcd for C_13_H_9_ClN_2_O_2_ (260.67): C, 59.90; H, 3.48; N, 10.75; Cl, 13.60. Found: C, 59.85; H, 3.42; N, 10.70; Cl, 13.58%.

#### 3.2.3. 5-(4-Bromophenylazo)salicylaldehyde (**4e**)

Yellow solid; Yield: 5.49 g (90%); mp 212–213 °C (from EtOH); IR (KBr) ν_max_: 3430 (OH), 1665 (C=O) cm^−1^; ^1^H NMR (DMSO-*d*_6_): δ = 7.18 (d, *J* = 9.0 Hz, 1H, ArH), 7.75–7.76 (m, 3H, ArH), 7.78 (d, *J* = 9.0 Hz, 1H, ArH), 8.06 (d, *J* = 9.0 Hz, 1H, ArH), 8.18 (d, *J* = 6.6 Hz, 1H, ArH), 10.36 (s, 1H, CHO), 11.53 (s, 1H, OH); MS (EI, 70 eV) *m*/*z* (rel. int.): 307 (M^+^ + 2, 0.5), 306 (M^+^ + 1, 0.4), 305 (M^+^, 1.8), 201 (3), 189 (4) 173 (2), 159 (3), 135 (3), 117 (18), 113 (19), 101 (17), 87 (20), 59, (100), 57 (12);Anal. Calcd for C_13_H_9_BrN_2_O_2_ (305.12): C, 51.17; H, 2.97; N, 9.18.; Br, 26.19. Found: C, 51.15; H, 2.95; N, 9.15; Br, 26.17%.

#### 3.2.4. 5-(3-Nitrophenylazo)salicylaldehyde (**4f**)

Orange solid; Yield: 4.99 g (92%); mp > 300 °C (from EtOH/DMF); IR (KBr) ν_max_: 3432 (OH), 1662 (C=O) cm^−1^; ^1^H NMR (DMSO-*d*_6_): δ = 7.20 (d, *J* = 8.7 Hz, 1H, ArH), 8.05–8.06 (m, 3H, ArH), 8.12 (d, *J* = 9.0 Hz, 1H, ArH), 8.23 (d, *J* = 8.8 Hz, 1H, ArH), 8.39 (d, *J* = 7.2 Hz, 1H, ArH), 10.37 (s, 1H, CHO), 11.72 (s, 1H, OH); MS (EI, 70 eV) *m*/*z* (rel. int.): 273 (M^+^ + 2, 0.18), 272 (M^+^ + 1, 0.1), 271 (M^+^, 0.02), 270 (M^+^ − 1, 0.02), 269 (M^+^ − 2, 0.03), 113 (12), 103 (9), 101(13) 87 (19), 69 (9), 59, (100), 57 (14), 55 (6); Anal. Calcd for C_13_H_9_N_3_O_4_ (271.22): C, 57.57; H, 3.34; N, 15.49. Found: C, 57.50; H, 3.32; N, 15.45%.

#### 3.2.5. 5-(4-Acetylphenylazo)salicylaldehyde (**4g**)

Orange solid; Yield: 5.09 g (95%); mp 174–176 °C (from EtOH); IR (KBr) ν_max_: 3436 (OH), 1700 (acetyl C=O), 1667 (C=O) cm^−1^; ^1^H NMR (DMSO-*d*_6_): δ = 2.64 (s, 3H, CH_3_), 7.20 (d, *J* = 9.0 Hz, 1H, ArH), 7.93 (d, *J* = 8.4 1H, ArH), 7.96 (d, *J* = 8.4 Hz, 1H, ArH), 8.10 (d, *J* = 9.0 Hz, 1H, ArH), 8.14–8.23 (m, 3H, ArH), 10.37 (s, 1H, CHO), 11.62 (s, 1H, OH); MS (EI, 70 eV) *m*/*z* (rel. int.): 270 (M^+^ + 2, 1), 269 (M^+^ + 1, 8), 268 (M^+^, 42), 149 (28), 121 (100) 104 (15), 93 (48), 91 (43), 76 (31), 65 (77), 57 (6), 52 (10), 51, (11), 50 (15); Anal. Calcd for C_15_H_12_N_2_O_3_ (268.26): C, 67.16; H, 4.51; N, 10.44. Found: C, 67.15; H, 4.50; N, 10.40%.

#### 3.2.6. 5-(4-Ethoxycarbonylphenylazo)salicylaldehyde (**4h**)

Orange solid; Yield: 5.48 g (92%); mp 170–172 °C (from EtOH); IR (KBr) ν_max_: 3435 (OH), 1706 (ester C=O), 1657 (C=O) cm^−1^; ^1^H NMR (DMSO-*d*_6_): δ = 1.35 (t, 3H, CH_3_), 4.31 (q, 2H, CH_2_), 7.19 (d, *J* = 8.7 Hz, 1H, ArH), 7.92 (d, *J* = 6.9 Hz, 1H, ArH), 7.96 (d, *J* = 7 Hz, 1H, ArH), 8.11 (d, *J* = 7.5 Hz, 1H, ArH), 8.13–8.22 (m, 3H, ArH), 10.37 (s, 1H, CHO), 11.61 (s, 1H, OH); MS (EI, 70 eV) *m*/*z* (rel. int.): 300 (M^+^ + 2, 0.6), 299 (M^+^ + 1, 4), 298 (M^+^, 20), 149 (57), 121 (100) 104 (31), 93 (49), 76 (46), 65 (99), 53 (15), 51 (14), 50 (27); Anal. Calcd for C_16_H_14_N_2_O_4_ (298.29): C, 64.42; H, 4.73; N, 9.39. Found: C, 64.40; H, 4.70; N, 9.30%.

#### 3.2.7. General Procedure for the Synthesis of 4-(5-Arylazo-2-hydroxystyryl)-1-methylpyridinium Iodide (**6a**–**i**)

**Method A** To a mixture of 1-methylpyridiniumiodide (1.17 g, 5 mmol) and arylazosalicylaldehyde **4a**–**i** (5 mmol) in absolute ethanol (40 mL) was added a catalytic amount (few drops) of piperidine. The reaction mixture was refluxed for 5 h and then cooled to room temperature. The precipitate formed was filtered off, washed with water and ethanol, and finally crystallized twice from the appropriate solvent to give the corresponding 1-methyl-4-(5-arylazo-2-hydroxystyryl)pyridinium iodide **6a**–**i**. The compounds **6a**–**i** prepared together with their physical constants are listed below.

**Method B** Diazotized analine derivatives **2g**–**i** which were prepared as previously described were added dropwise with stirring to a cooled solution (0 °C) of compouned **7** (1.06 g, 5 mmol) in aq. ethanol (5 mL H_2_O in 45 mL EtOH) containing 1 g of NaOH. Following the addition, the pH of the reaction mixture was adjusted to 7, the precipitate was filtered and recrystallised from the appropriate solvent to give the respective 4-(5-arylazo-2-hydroxystyryl)-1-methylpyridinium iodide. By this method the compouneds **6g**–**i** were prepared and proved identical in all respects (mp, mixed mp and IR) with the respective dyes prepared by method A.

#### 3.2.8. 4-(2-Hydroxy-5-(4-methoxyphenylazo)styryl)-1-methylpyridinium Iodide (**6a**)

Dark yellow solid; Yield: 2.01 g (85%); mp 114–115 °C (from EtOH); IR (KBr) ν_max_: 3438 (OH), 2931 (aliphatic CH),1599 (aromatic C=C) cm^−1^; ^1^H NMR (DMSO-*d*_6_): δ = 3.83 (s, 3H, OCH_3_), 4.17 (s, 3H, NCH_3_), 6.77 (s, 1H, OH), 7.06–7.08 (m, 3H, ArH), 7.70–7.77 (m, 4H, ArH), 8.04–8.13 (m, 4H, ArH), 8.64–8.66 (d, 2H, *J* = 6 Hz, olefinic H); MS (EI, 70 eV) *m*/*z* (rel. int.): 475 (M^+^ + 2, 27), 474 (M^+^ + 1, 34), 473 (M^+^, 47), 458 (36), 419 (41), 385 (41), 354 (48), 319 (41), 254 (100), 224 (41), 203 (47), 192 (43), 188 (42), 174 (45), 108 (42), 104 (45), 95 (45), 68 (46), 57 (90). Anal. Calcd for C_21_H_20_IN_3_O_2_ (473.31): C, 53.29; H, 4.26; N, 8.88. Found: C, 53.25; H, 4.20; N, 8.85%.

#### 3.2.9. 4-(2-Hydroxy-5-(4-methylphenylazo)styryl)-1-methylpyridinium Iodide (**6b**)

Yellow solid; Yield: 1.83 g (80%); mp 152–154 °C (from EtOH); IR (KBr) ν_max_: 3423 (OH), 3038 (aromatic CH), 1592 (aromatic C=C) cm^−1^; ^1^H NMR (DMSO-*d*_6_): δ = 2.36 (s, 3H, CH_3_), 4.16 (s, 3H, NCH_3_), 6.66 (s, 1H, OH),7.29–7.32 (m, 3H, ArH), 7.63–7.78 (m, 4H, ArH), 8.04 (d, *J* = 8 Hz, 2H, ArH), 8.12 (d, *J* = 8 Hz, 2H, ArH), 8.61–8.63 (d, 2H, *J* = 6 Hz, olefinic H); MS (EI, 70 eV) *m*/*z* (rel. int.): 458 (M^+^ + 1, 22), 457 (M^+^, 28), 436 (26), 424 (29), 408 (28), 381 (31), 377 (29),363 (29), 354 (34), 348 (29), 330 (29), 317 (32), 298 (43), 276 (39), 260 (34), 198 (34), 135 (59), 120 (100), 106 (20), 64 (39). Anal. Calcd for C_21_H_20_IN_3_O (457.31): C, 55.15; H, 4.41; N, 9.19. Found: C, 55.10; H, 4.35; N, 9.15%.

#### 3.2.10. 4-(2-Hydroxy-5-phenylazostyryl)-1-methylpyridinium Iodide (**6c**)

Yellow solid; Yelid: 1.75 g, (78%); mp 112–113 °C (from EtOH); IR (KBr) ν_max_: 3418 (OH), 3041 (aromatic CH), 1607 (aromatic C=C) cm^−1^; ^1^H NMR (DMSO-*d*_6_): δ = 4.26 (s, 3H, NCH_3_), 6.69 (s, 1H, OH), 7.53–7.61 (m, 5H, ArH), 7.66–7.89 (m, 3H, ArH), 8.11(d, *J* = 8.2 Hz, 2H, ArH), 8.25 (d, *J* = 8.4 Hz, 2H, ArH), 8.84–8.86 (d, 2H, *J* = 6 Hz, olefinic H); MS (EI, 70 eV) *m*/*z* (rel. int.): 444 (M^+^ + 1, 12), 443 (M^+^, 21), 430 (31), 414 (35), 400 (25), 376 (39), 368 (34), 363 (26), 353 (44), 344 (32), 330 (29), 317 (25), 297 (45), 275 (33), 261 (39), 198 (35), 135 (59), 120 (58), 105 (88), 65 (45), 59 (100), 57 (11). Anal. Calcd for C_20_H_18_IN_3_O (443.28): C, 54.19; H, 4.09; N, 9.48. Found: C, 54.10; H, 4.00; N, 9.45%.

#### 3.2.11. 4-(2-Hydroxy-5-(3-chlorophenylazo)styryl)-1-methylpyridinium Iodide (**6d**)

Orange solid; Yield: 2.26 g, (95%); mp 167–168 °C (from EtOH); IR (KBr) ν_max_: 3430 (OH), 3040 (aromatic CH), 2925 (aliphatic CH), 1597 (aromatic C=C) cm^−1^; ^1^H NMR (DMSO-*d*_6_): δ = 4.26 (s, 3H, NCH_3_), 6.72 (s, 1H, OH), 7.36–7.40 (m, 3H, ArH), 7.41–7.80 (m, 4H, ArH), 8.05–8.11 (m, 4H, ArH), 8.66–8.68 (d, 2H, *J* = 6 Hz, olefinic H); MS (EI, 70 eV) *m*/*z* (rel. int.): 479 (M^+^ + 2, 19), 478 (M^+^ + 1, 20), 477 (M^+^, 19), 435 (23), 418 (29), 407 (20), 389 (20), 379 (28), 350 (22), 338 (38), 327 (20), 313 (33), 304 (24), 291 (28), 278 (23), 267 (32), 245 (23), 228 (29), 216 (22), 209 (24), 192 (21), 171 (20), 162 (31), 137 (23), 109 (35), 98 (48), 73 (43), 69 (68),57 (100). Anal. Calcd for C_20_H_17_ClIN_3_O (477.73): C, 50.28; H, 3.59; Cl, 7.42; N, 8.80. Found: C, 450.20; H, 3.55; Cl, 7.40 N, 8.80%.

#### 3.2.12. 4-(2-Hydroxy-5-(4-bromophenylazo)styryl)-1-methylpyridinium Iodide (**6e**)

Orange; Yield: 2.14 g, (82%); mp 211–212 °C (from EtOH); IR (KBr) ν_max_: 3426 (OH), 2995 (aliphatic CH), 1589 (aromatic C=C) cm^−1^; ^1^H NMR (DMSO-*d*_6_): δ = 4.09 (s, 3H, NCH_3_), 6.26 (s, 1H, OH), 7.60–7.64 (m, 3H, ArH), 7.78–7.92 (m, 4H, ArH), 7.98–8.03 (m, 4H, ArH), 8.48–8.50 (d, 2H, *J* = 6 Hz, olefinic H); MS (EI, 70 eV) *m*/*z* (rel. int.): 525 (M^+^ + 3, 32), 524 (M^+^ + 2, 47), 519 (44), 518 (38), 517 (42), 512 (44), 509 (42),458 (40), 432 (40), 375 (48), 362 (40), 355 (44), 342 (41), 323 (43), 299 (41), 287 (50), 279 (54), 271 (43), 223 (41), 211 (44), 187 (55), 173 (84), 142 (42), 127 (45), 95 (47), 65 (100), 60 (44), 54 (54), 52 (61). Anal. Calcd for C_20_H_17_BrIN_3_O (522.18): C, 46.00; H, 3.28; N, 8.05. Found: C, 46.02; H, 3.25; N, 8.01%.

#### 3.2.13. 4-(2-Hydroxy-5-(3-nitrophenylazo)styryl)-1-methylpyridinium Iodide (**6f**)

Reddish yellow solid; Yield: 1.58 g, (65%), mp 280–282 °C (from EtOH); IR (KBr) ν_max_: 3434 (OH), 1594 (aromatic C=C) cm^−1^; ^1^H NMR (DMSO-*d*_6_): δ = 4.12 (s, 3H, NCH_3_), 6.25 (s, 1H, OH),7.66–7.73 (m, 3H, ArH), 7.80–7.96 (m, 4H, ArH), 7.99–8.34 (m, 4H, ArH), 8.54–8.56 (d, 2H, *J*=6 Hz, olefinic H); MS (EI, 70 eV) *m*/*z* (rel. int.): 489 (M^+^ +1, 15), 488 (M^+^, 17), 448 (25), 431 (19), 405 (19), 381 (29), 349 (21),341 (23), 328 (34), 301 (22), 274 (25), 242 (20), 236 (23), 198 (21), 144 (19), 120 (100), 106 (69), 90 (14), 59 (14). Anal. Calcd for C_20_H_17_IN_4_O_3_ (488.28): C, 49.20; H, 3.51; N, 11.47. Found: C, 49.10; H, 3.50; N, 11.42%.

#### 3.2.14. 4-(2-Hydroxy-5-(4-acetylphenylazo)styryl)-1-methylpyridinium Iodide (**6g**)

Orange solid; Yield 2.06 g, (85%); mp 174–175 °C (from EtOH); IR (KBr) ν_max_: 3429 (OH), 3040 (aromatic CH), 1590 (aromatic C=C) cm^−1^; ^1^H NMR (DMSO-*d*_6_): δ = 2.61 (s, 3H, COCH_3_), 4.19 (s, 3H, NCH_3_), 6.72 (s, 1H, OH), 7.74–7.78 (m, 3H, ArH), 7.80–8.09 (m, 4H, ArH), 8.13–8.14 (m, 4H, ArH), 8.67–8.69 (d, 2H, *J* = 6 Hz, olefinic H); MS (EI, 70 eV) *m*/*z* (rel. int.): 486 (M^+^ + 1, 12), 485 (M^+^, 18), 482 (12), 460 (13), 452 (13), 428 (15), 422 (18), 403 (16), 377 (16), 362 (20), 339 (18), 324 (16), 307 (16), 283 (17), 276 (18), 264 (17), 252 (21), 232 (17), 196 (24), 186 (15), 173 (18), 158 (21), 155 (22), 120 (64), 108 (17), 93 (100), 78 (39), 66 (32), 65 (56). Anal. Calcd for C_22_H_20_IN_3_O_2_ (485.32): C, 54.45; H, 4.15; N, 8.66. Found: C, 54.44; H, 4.10; N, 8.60%.

#### 3.2.15. 4-(2-Hydroxy-5-(4-Ethoxycarbonylphenylazo)styryl)-1-methylpyridinium Iodide (**6h**)

Reddish yellow solid; Yield: 2.26 g, (88%); mp 170–172 °C (from EtOH); IR (KBr) ν_max_: 3407 (OH), 3055 (aromatic CH), 2923 (aliphatic CH), 1700 (C=O), 1589 (aromatic C=C) cm^−1^; ^1^H NMR (DMSO-*d*_6_): δ = 1.33 (t, 3H, *J* = 6.9 Hz, CH_3_), 4.10 (s, 3H, NCH_3_), 4.33 (q, 2H, *J* = 6.9 Hz, CH_2_), 6.31 (s, 1H, OH), 7.65–7.69 (m, 3H, ArH), 7.71–7.84 (m, 4H, ArH), 7.92–8.03 (m, 4H, ArH), 8.50–8.52 (d, 2H, *J* = 6 Hz, olefinic H); MS (EI, 70 eV) *m*/*z* (rel. int.): 517 (M^+^ + 2, 45), 516 (M^+^ + 1, 100), 515 (M^+^, 61), 506 (70), 477 (61), 443 (63), 430 (68), 383 (63), 355 (61), 336 (68), 331 (62), 327 (65), 321 (66), 295 (94), 283 (68), 280 (68), 273 (61), 249 (84), 224 (68), 183 (61), 109 (61), 71 (61), 65 (63). Anal. Calcd for C_23_H_22_IN_3_O_3_ (515.34): C, 53.60; H, 4.30; N, 8.15. Found: C, 53.55; H, 4.28; N, 8.10%.

#### 3.2.16. 4-(2-Hydroxy-5-(4-nitrophenylazo)styryl)-1-methylpyridinium Iodide (**6i**)

Red solid; Yield 2.19 g, (90%); mp 304 °C (from EtOH/dioxane); IR (KBr) ν_max_: 3434 (OH), 1597 (aromatic C=C) cm^−1^; ^1^H NMR (DMSO-*d*_6_): δ = 4.16 (s, 3H, NCH_3_), 6.47 (s, 1H, OH), 7.73–7.77 (m, 3H, ArH), 7.81–8.05 (m, 4H, ArH), 8.29–8.32 (m, 4H, ArH), 8.61–8.63 (d, 2H, *J* = 6 Hz, olefinic H); MS (EI, 70 eV) *m*/*z* (rel. int.): 490 (M^+^ + 2, 53), 489 (M^+^ + 1, 68), 485 (60), 341 (70), 323 (61), 307 (60), 288 (71), 4273 (71),233 (100), 189 (71), 187 (83), 160 (67), 123 (79), 101 (68), 94 (67), 78 (76), 55 (66). Anal. Calcd for C_20_H_17_IN_4_O_3_ (488.28): C, 49.20; H, 3.51; N, 11.47. Found: C, 49.15; H, 3.49; N, 11.45%.

### 3.3. pK′s and pK*′s Determination of the Prepared Azo Merocyanine Dyes **6a**–**i**

The dissociation constants pKa′s of compounds **6** were calculated spectrophotometrically in 80% dioxane-water mixture at 27 ± 0.1 °C and ionic strength (KNO_3_) 0.1. The Orion pH meter 420A (Cole-Parmer, Vernon Hills, IL, USA) fitted with combined glass electrode type 518635 was used for measurement of pH values. The instrument was accurate to ±0.01 pH unit. It was calibrated using two Beckman standard buffer solutions of pH 4.01 and 7.00. The pH meter readings (B) recorded in dioxane-water solutions were converted to the concentration of hydrogen ion [H^+^] by means of the equation of van Uitert and Haas [16] namely:

−log [H^+^] = B + log UH

where log UH is the correction factor for the solvent composition and ionic strength used for which B is read. The value of log UH was determined by recording the pH values for a series of hydrochloric acid and sodium chloride solutions such that the ionic strength was 0.1 in 20% (*v*/*v*) water-dioxane mixture at 27 °C. The value of log UH was calculated and found to be −0.48. The experimental procedures obey in the evaluation of acid dissociation constants pKa and their calculations from the A-pH data and are as literature described in the literature [17]. The pKa values were reproducible to ±0.02 pKa unit. The results are listed in Table 2.

## 4. Conclusions

In conclusion, the pK and pK* values of the studied hydroxyazo compounds decreases in the following order **6a** > **6b** > **6c** > **6e** > **6d** > **6h** > **6g** > **6f** > **6i**, alsothe foregoing data indicate that the studied compounds exist in one tautomeric form namely the hydroxyazo form **6A** in both ground and excited states and solvatochromic behavior of the title compounds are studied in eight organic solvent using Kamlet-Taft equation to increases the opportunity uses of these compounds as potential photoprobe.

## Figures and Tables

**Table 1:** UV Spectra of 4-(5-arylazo-2-hydroxystyryl)-1-methylpyridinium iodide **6a**–**i** in ethanol.

Compound No.	λ_max_ nm (EtOH) (log ε)	Compound No.	λ_max_ nm (EtOH) (log ε)
**6a**	542 (4.01), 421 (4.16), 361 (4.25)	**6f**	525 (4.17), 443 (4.11), 347 (4.11)
**6b**	538 (3.95), 411 (4.06), 355 (4.12)	**6g**	535 (4.48), 459 (4.42), 350 (4.45)
**6c**	530 (3.81), 412 (4.00), 353 (4.15)	**6h**	528 (4.48), 452 (4.43), 345 (4.32)
**6d**	530 (4.18), 428 (4.17), 353 (4.16)	**6i**	543 (3.45), 484 (3.38), 347 (3.14)
**6e**	534 (4.51), 433 (4.50), 352 (4.43)		

**Table 2:** Acid dissociation constants pK and pK* of 4-(5-arylazo-2-hydroxystyryl)-1-methylpyridinium iodide **6a**–**i**.

Compd. No.	σ_X_	$\sigma_{x}^{-}$	pK	λ_max_^a^	λ_max_^b^	Δν cm^−1^	pK*
**6a**	−0.27	−0.27	7.92	353	537	9706	−12.46
**6b**	−0.17	−0.17	7.70	352	529	9505	−12.26
**6c**	0	0	7.57	350	524	9487	−12.35
**6d**	0.37	0.37	7.18	344	520	9838	−13.48
**6e**	0.23	0.23	7.38	346	525	9854	−13.31
**6f**	0.71	0.71	6.86	346	527	9926	−13.98
**6g**	0.5	0.87	6.94	347	525	9771	−13.58
**6h**	0.45	0.68	7.09	349	533	9891	−13.68
**6i**	0.78	1.28	6.75	363	575	10157	−14.58

^a^ In acid medium; ^b^ in alkaline medium; ±*s* = 0.04.

**Table 3:** Electronic spectral data of 4-(5-arylazo-2-hydroxystyryl)-1-methylpyridinium iodide **6a**–**i** in various solvents.

Compd. No.		λ_max_ nm (log ε)						
	MeCN	*n*-BuOH	HCCl_3_	DMF	DMSO	MeOH	EtOH	*n*-PrOH
**6a**	578 (3.84),	547 (4.08),	665 (3.66),	560 (3.11), 357 (4.36)	588 (4.17),	526 (3.34), 354 (4.33)	542(4.01),	549 (3.85),
	434 (3.96),	426 (4.15),	439 (3.99),		459 (4.07),		421(4.16),	425 (4.08),
	354 (4.22)	567 (4.18)	361 (4.21)		350 (4.09)		361(4.25)	363 (4.23)
**6b**	572 (3.99),	543 (4.15),	653 (3.9),	749 (2.67),	582 (4.34),	511(3.82),	538(3.95),	547 (3.98),
	435 (3.96),	420 (4.14),	432 (4.02),	556 (3.57),	460 (4.19),	411 (4.12),	411(4.06),	416 (4.09),
	346 (4.16)	362 (4.13)	353 (4.13)	347(4.36)	342 (4.15)	347 (4.30)	355(4.12)	357(4.18);
**6c**	575 (3.04),	538 (3.94),	587 (2.60),	587 (4.29),	578 (3.94),	513 (3.58),	530(3.81),	538 (3.77),
	519 (3.15),	423 (3.97),	418 (3.85),	460 (4.15),	457 (3.86),	402 (3.97),	412(4.00),	410 (4.08),
	344 (4.48)	359 (4.02)	348 (4.16)	347 (4.08)	344 (4.07)	348 (4.17)	353(4.15)	350 (4.26)
**6d**	564 (4.21),	536 (3.94),	643 (3.82),	581 (4.35),	574 (4.38),	508 (3.85),	530(4.18),	538 (3.98),
	455 (4.06),	432 (3.89),	429 (3.98),	475 (4.09),	475 (4.13),	404 (4.17),	428(4.17),	430 (4.02),
	343 (4.24)	356 (3.83)	356 (4.11)	338 (3.97)	339 (4.02)	347 (4.29)	353(4.16)	353 (4.06)
**6e**	564 (4.69),	538 (4.55),	647 (4.67),	551 (4.18), 347 (4.62)	567 (4.76),	509 (4.27),	534(4.51),	543 (4.53),
	458 (4.54),	438 (4.52),	455 (4.48),		474 (4.55),	405 (4.49),	433(4.50),	432 (4.53),
	340 (4.39)	353 (4.41)	392 (4.35)		338 (4.37)	348 (4.60)	352(4.43)	353 (4.47)
**6f**	561(4.28),	532 (4.23),	628 (4.21)	577 (4.47),	573 (4.41),	507 (4.02),	525(4.17),	533 (4.19),
	469 (4.09),	450 (4.18),	459 (4.04),	483 (4.21),	483 (4.17),	414 (4.11),	443(4.11),	444 (4.15),
	340 (4.08)	349 (4.14)	362 (4.04)	337 (4.14)	336 (4.09)	345 (4.25)	347(4.11)	350 (4.18)
**6g**	571 (4.43),	544 (4.55),	637 (4.27),	564 (4.59),	586 (4.78),	507 (4.02),	535(4.48),	545 (4.44),
	478 (4.25),	466 (4.48),	461 (4.27),	477 (4.40),	492 (4.49),	414 (4.11),	459 (4.42),	456 (4.40),
	348 (4.48)	351 (4.38)	359 (4.51)	343 (4.61)	340 (4.41)	345 (4.25)	350(4.45)	351 (4.45)
**6h**	569 (4.52),	539 (4.66),	639 (4.6),	533 (4.52),	584 (4.75),	516 (4.25),	528(4.48),	542 (4.48),
	476 (4.47),	459 (4.46),	470 (4.41),	453 (4.46),	489 (4.45),	413 (4.39),	452(4.43),	454 (4.43),
	344 (4.32)	346 (4.32)	371 (4.25)	344 (4.33)	333 (4.28)	349 (4.55)	345(4.32)	346 (4.36)
**6i**	591 (3.67),	548 (3.45),	648 (3.17), 506 (3.44)	619 (4.67),	618 (4.48),	521 (3.94),	543(3.45),	550 (3.49),
	503 (3.47),	478 (3.38),		508 (4.30),	504 (4.12),	420 (4.04),	484(3.38),	488 (3.41),
	352 (3.47)	346 (3.36)		342 (4.26)	336 (4.07)	350 (4.24)	347(3.14)	344 (3.44)

**Table 4:** Solvent parameters.

Solvents	π*	Β	Α
Acetonitrile	0.75	0.31	0.19
1-Butanol	0.47	0.88	0.79
Chloroform	0.58	0	0.44
DMF	0.88	0.69	0
DMSO	1	0.76	0
Ethanol	0.54	0.77	0.83
Methanol	0.6	0.62	0.93
1-Propanol	0.52	0.83	0.78

**Table 5:** Regression fits to solvatochromic parameters (Equation (1)).

Compd. No.	*υ*_o_ (10^3^·cm^−1^)	*s*	*b*	*a*	*R* ^1^	±*S* ^2^
**6a**	24.38	4.778	−0.752	1.588	0.987	0.092
**6b**	24.819	5.158	−0.825	1.471	0.982	0.136
**6c**	13.738	3.410	0.390	3.630	0.987	0.197
**6d**	19.389	3.103	−1.782	4.769	0.993	0.202
**6e**	22.80	5.448	2.292	1.136	0.810	0.974
**6f**	16.62	5.23	−1.25	5.36	0.991	0.208
**6g**	26.462	2.428	0.792	0.216	0.983	0.114
**6h**	24.58	3.217	2.047	0.483	0.921	0.438
**6i**	16.544	7.699	8.838	1.542	0.896	1.925

^1^ Correlation coefficient; ^2^ Standard error of the estimate.

**Table 6:** Contribution percentages of solvatochromic parameters π*, β and α.

Compod. No.	π* (%)	β (%)	α (%)
**6a**	67.12	10.56	22.31
**6b**	69.19	11.07	19.73
**6c**	45.89	5.24	48.86
**6d**	32.14	18.45	49.39
**6e**	61.38	25.82	12.79
**6f**	44.17	10.55	45.27
**6g**	70.66	23.05	6.28
**6h**	55.97	35.61	8.40
**6i**	42.58	48.88	8.53
